# Aberrant static and dynamic functional connectivity of auditory processing in migraine: A millisecond-scale magnetoencephalography study

**DOI:** 10.7555/JBR.39.20250487

**Published:** 2026-09-28

**Authors:** Hongxing Liu, Yuanwen Yu, Di Wu, Qiqi Chen, Yingfan Wang, Minghao Li, Xiaoshan Wang

**Affiliations:** 1 Department of Neurology, the Affiliated Brain Hospital of Nanjing Medical University, Nanjing, Jiangsu 210029, China; 2 MEG Center, the Affiliated Brain Hospital of Nanjing Medical University, Nanjing, Jiangsu 210029, China

**Keywords:** migraine, magnetoencephalography, functional connectivity, auditory task, interictal period, dynamic functional connectivity

## Abstract

This study aimed to characterize frequency- and latency-dependent network alterations in migraine using magnetoencephalography (MEG)-based static and dynamic functional connectivity analyses during auditory stimulation. Thirty interictal patients with migraine and 30 healthy controls underwent whole-head MEG recordings during a lateralized auditory task. Connectivity was estimated using corrected amplitude-envelope correlation across seven predefined frequency bands spanning 2–120 Hz. Group differences were assessed using two-sided independent-samples *t*-tests, with Bonferroni correction for static analyses and false discovery rate correction for dynamic analyses. Static differences were confined to low-gamma (30–59 Hz), high-gamma (60–89 Hz), and ripple (90–120 Hz) bands. Static functional connectivity was significantly higher in patients with migraine (Cohen's *d* = 1.40–1.48 for the illustrated connections). No static connections survived correction in the delta, theta, alpha, or beta bands. Dynamic analyses revealed early gamma/ripple increases and additional low-frequency changes across time windows. These findings suggest altered auditory network organization in interictal migraine. Their potential relevance to sensory processing requires further testing in independent cohorts, including direct assessment of auditory symptoms.

## Introduction

Migraine is a common neurological disorder affecting approximately 1.16 billion people worldwide. It is the second most common neurological condition after tension-type headache and the second leading cause of disability globally, surpassed only by low back pain^[1]^. The one-year prevalence of migraine is around 15% in adults and 7% in children, with women affected at roughly three times the rate of men. The disorder usually peaks between ages 35 and 39, and 75% of patients experience their first episode before age 35^[2]^. According to the Global Burden of Disease 2021 data, migraine accounts for 43.4 million years lived with disability (YLDs), constituting a substantial portion of global YLDs and remaining the leading cause of disability among young women^[3]^. The International Classification of Headache Disorders, 3rd Edition (ICHD-3), classifies migraine into three main subtypes: migraine without aura (70%–80% of cases), migraine with aura (20%–30%), and chronic migraine^[4]^. Typical symptoms include a moderate to severe unilateral pulsating headache lasting 4–72 h, often accompanied by nausea, vomiting, photophobia, and phonophobia, with aura lasting 5–60 min^[5]^. Migraine substantially impairs quality of life, resulting in reduced participation in work and social activities and an elevated risk of anxiety and depression^[6]^.

Phonophobia, a heightened sensitivity to sound, often referred to as auditory hyperesthesia, is a common symptom of migraine, affecting 70%–80% of cases during acute attacks and often persisting into the interictal period^[7]^. Studies report interictal phonophobia prevalence rates ranging from 63.6% to 86.7%^[8]^. The underlying mechanisms involve central sensitization of brainstem auditory pathways, increased brainstem auditory evoked potentials, impaired habituation, and reduced sensory thresholds, all of which may be linked to neuroinflammation and cortical spreading depression (CSD)^[9]^. This symptom can impair social and occupational functioning, contributing to increased psychological distress.

The pathophysiology of migraine is multifactorial, involving genetic influences, neuronal hyperexcitability, vascular dysregulation, and neuroinflammation. A central mechanism is the activation of the trigeminovascular system and the release of calcitonin gene-related peptide^[10]^, while CSD is widely recognized as the neurophysiological basis of migraine aura^[11]^. Additionally, both the hypothalamus and central sensitization are implicated in migraine chronification and symptom modulation^[12]^. Advanced neuroimaging techniques, such as functional magnetic resonance imaging (fMRI), diffusion tensor imaging, magnetic resonance spectroscopy, and positron emission tomography, have revealed interictal abnormalities in hypothalamic-brainstem networks, white matter microstructural alterations, and neurotransmitter imbalances^[13]^. Furthermore, electrophysiological studies using electroencephalography (EEG) and magnetoencephalography (MEG) have demonstrated interictal increases in theta-band activity, decreased alpha/beta connectivity, abnormal habituation, and evidence of central sensitization^[14]^.

Migraine is often linked to auditory disturbances such as phonophobia and tinnitus, reflecting dysfunctional auditory processing^[15]^. Specific deficits include impaired auditory gap detection, reduced temporal resolution, and abnormal brainstem responses^[16]^. Event-related potential studies have demonstrated abnormalities in mismatch negativity and P300 components^[17]^. Although these abnormalities have been investigated using auditory behavioral tests, neuroimaging (*e.g.*, fMRI), and self-report questionnaires, methodological heterogeneity across studies has contributed to inconsistent results^[18]^.

MEG is a neuroimaging technique with a temporal resolution of a few milliseconds, capable of measuring the neuromagnetic fields generated in the brain. It avoids the vascular artifacts seen in fMRI and offers better spatial resolution than EEG. These properties make it ideal for studying migraine parameters such as CSD and network fluctuations^[19]^. Noninvasive, silent, and radiation-free, MEG can also be used to identify the source of activity and to develop potential biomarkers. It has been employed in both interictal and ictal states, and is often used alongside computational modeling to relate the findings to cognitive functions^[20]^.

In auditory paradigms, MEG highlights event-related fields and enables source localization, revealing alterations in attentional networks in individuals with migraine^[21]^. For example, Masson *et al*^[22]^ observed increased evoked responses to both target and distractor sounds in interictal patients, accompanied by hyperactivation of the right temporoparietal junction, a key node of the ventral attention network, suggesting heightened susceptibility to auditory stimuli. Another study reported decreased mismatch negativity amplitude during attacks, interpreted as increased inhibition, though functional connectivity was not directly assessed^[23]^. These findings indirectly suggest network reorganization in migraine.

Functional connectivity analysis of envelope coherence, especially using the corrected amplitude-envelope correlation (AEC-c), is particularly suited for analyzing MEG and EEG data. This approach involves band-pass filtering neural signals into specific frequency bands, deriving amplitude envelope estimators with the Hilbert transform, and calculating Pearson's correlation for these envelope coherences between pairs of brain regions. The main drawback of this approach stems from artifacts, especially spatial leakage caused by volume conduction or common field distributions in noninvasive recordings like MEG. To address this, AEC-c employs pairwise orthogonalization, where signals are regressed against each other to isolate genuine interactions, resulting in more accurate and interpretable connectivity estimates that are typically normalized to a [0, 1] range. This technique is better suited for detecting frequency-specific oscillatory dynamics, as it allows the study of how brain networks synchronize functionally in terms of amplitude rather than phase, which often suffers from noise or zero-lag correlations^[24]^. In neurological disorders such as migraine, where the interictal brain states involve minor dysregulations of cortical excitability or network integration, AEC-c has demonstrated significant advantages. For example, it has been used to identify frequency-specific abnormalities, such as increased path lengths in alpha-band networks during the interictal phase of migraine without aura, suggesting impaired information integration across cortical regions^[25]^. Its ability to detect dynamic, band-specific activity changes provides deeper insight into the pathophysiological features of migraine, such as hyperexcitability and deficits in cross-modal sensory processing, making it more effective than static measurements and aiding in biomarker discovery for targeted interventions^[26]^.

By examining static and dynamic functional connectivity during lateralized auditory stimulation, this study aimed to characterize the frequency and temporal organization of auditory networks in interictal migraine. The findings may help identify candidate electrophysiological signatures of altered auditory processing, which require validation in independent cohorts. Their diagnostic or therapeutic utility was not evaluated in this study.

## Materials and methods

### Subjects

Thirty patients with migraine (mean age: 31.07 [± 6.55] years; range: 16–40 years) and 30 age- and sex-matched healthy controls were enrolled in this study. The patient group included six individuals with migraine with aura (MwA) and 24 with migraine without aura (MwoA), all diagnosed based on ICHD-3^[4]^. All patients were studied in the strictly interictal period (≥ 72 h from last/next attack, verified by diary and telephone follow-up; mean 6.8 [± 4.2] days from last attack). An exploratory comparison between MwA and MwoA did not detect significant differences; however, the small MwA subgroup (*n* = 6) precluded reliable subtype-specific inference. Healthy controls had no personal or first-degree family history of migraine or other primary headache disorders. All participants were right-handed.

Exclusion criteria for all participants included: (1) presence of ferromagnetic implants (*e.g.*, stents, pacemakers); (2) inability to stay still or comply with experimental procedures; and (3) contraindications or safety concerns precluding MRI scanning, such as claustrophobia or pregnancy. All participants had normal hearing and motor function and provided written informed consent before the study. Prior to enrollment, each participant was informed about the entire process, the associated risks, and the compensation offered. Informed consent was obtained from all individuals. The study protocol was reviewed and approved by the Institutional Review Board of the Affiliated Brain Hospital of Nanjing Medical University (Approval No. 2024-KY090-01).

### Auditory task

All subjects lay supine in the MEG scanner. Auditory stimuli consisted of 500 Hz, 30-ms square-wave tones, randomly delivered monaurally *via* plastic tubes or headphones. The entire stimulation involved 160 square-wave tones, with 80 presentations per ear. Subjects were instructed to immediately extend the index finger ipsilateral to the stimulated ear upon hearing the auditory stimulus and lightly press the sensor as prompted^[27–29]^. They were required to keep other limbs still and focus on a target. Pressing the button sent a signal from the response box to the MEG system. Stimulus presentation and response recording were performed using BrainX software (Cincinnati Children's Hospital Medical Center, Cincinnati, OH, USA), which is based on DirectX (Microsoft Corporation, Redmond, WA, USA). The entire testing process lasted approximately 15 min. Mean reaction time across participants was 412 (± 87) ms (range 268–689 ms; ***Supplementary Table 1***). The primary 0–200-ms interval preceded the earliest recorded button press and was selected to reduce contamination from overt motor execution.

### MEG signal acquisition

MEG data were acquired in the central magnetically shielded room at the Affiliated Brain Hospital of Nanjing Medical University, using the CTF 275-channel whole-head MEG system (VSM MedTech Systems Inc., Coquitlam, BC, Canada). Before data collection, participants removed all metal objects from their bodies, and then fiducial electromagnetic coils were placed on the nasion and the left and right preauricular points. A digitizer was used to determine the subject's coordinates, with the axes defined as follows: the X-axis as the line connecting the bilateral preauricular points (positive to the right), the Y-axis forward from the nasion, and the Z-axis perpendicular to the X-Y plane (positive upward). This setup was used for MEG localization. During recording, subjects lay supine in the MEG scanner with arms resting at their sides and were instructed to remain as still as possible. The MEG sampling rate was 6000 Hz per channel, and the data collection window was 3000 ms per trial (including 2000 ms pre-trigger or pre-finger press). Data were recorded after noise reduction using third-order gradients and nonlinear filtering. Head position was monitored at both the beginning and end of each run; if head movement exceeded 5 mm during these checks, the dataset was excluded, and the run was repeated.

### MRI acquisition and source reconstruction

Structural MRI was acquired using a 3.0-T GE Discovery MR750 scanner (GE Healthcare, Milwaukee, WI, USA) with an eight-channel head coil and an axial three-dimensional T1-weighted BRAVO sequence. Acquisition parameters were as follows: repetition time, 8.168 ms; echo time, 3.184 ms; inversion time, 450 ms; flip angle, 12°; voxel size, 0.5 × 0.5 × 1.0 mm³; matrix, 512 × 512; and 184 slices. Individual cortical surfaces were reconstructed from the T1-weighted images using FreeSurfer (version 7.4.1; Laboratory for Computational Neuroimaging, Athinoula A. Martinos Center for Biomedical Imaging, Massachusetts General Hospital, Boston, MA, USA). MEG and MRI data were coregistered using the nasion, left and right preauricular points, and additional head-shape points. The cortical surface comprised approximately 15000 vertices. An overlapping-spheres forward model and depth-weighted minimum-norm estimation were used, with source orientations constrained normal to the cortical surface and a signal-to-noise ratio of 3. Noise covariance was estimated from the −500 to −0.9 ms baseline. Source activity was parcellated into 68 cortical regions using the Desikan–Killiany atlas, and the representative time series for each parcel was extracted using the first principal component.

### Data analysis

MEG data were processed using Brainstorm software^[30]^. Continuous recordings were initially band-pass filtered at 0.2–1000 Hz using even-order linear-phase Kaiser-window finite impulse response filters with 60-dB stop-band attenuation. Filter orders were estimated using kaiserord, and group-delay compensation was applied. Second-order zero-phase infinite impulse response (IIR) notch filters were applied at 50, 100, 150, 200, and 250 Hz.

Bad channels and artifact-contaminated segments were identified by visual inspection and excluded. Ocular and cardiac artifacts were addressed using signal-space projection (SSP). Eye blinks were detected by thresholding vertical electrooculography (EOG) recordings; heartbeat-related artifacts were identified from the MEG data, and the corresponding projection components were selected following inspection.

The recordings were segmented into stimulus-related epochs. Baseline correction used the pre-stimulus interval from −500 ms to −0.9 ms. Epochs containing prominent residual artifacts were rejected by visual inspection. Each participant completed 160 trials, with 80 presented to each ear; approximately 98% of trials were retained, corresponding to approximately 78–79 valid trials per stimulation side. Artifact-free epochs were averaged separately for left- and right-ear stimulation before source reconstruction and connectivity analysis. Connectivity was not calculated separately for each trial and then averaged.

The primary frequency-specific analyses used signals restricted to 1–120 Hz, which were decomposed into delta (2–4 Hz), theta (5–7 Hz), alpha (8–12 Hz), beta (15–29 Hz), low-gamma (30–59 Hz), high-gamma (60–89 Hz), and ripple (90–120 Hz) bands. A separate exploratory broadband network-based statistic (NBS) analysis used 1–1000-Hz signals and is reported in ***Supplementary Figs. 1*** and ***2*** and ***Supplementary Table 2***.

### Functional connectivity

Functional connectivity was computed on source-reconstructed signals parcellated into 68 cortical regions according to the Desikan–Killiany atlas. Both static and dynamic functional connectivity were calculated using AEC-c within seven canonical frequency bands described above.

Amplitude envelopes were obtained from band-pass filtered source signals using the Hilbert transform in Brainstorm^[30]^. Envelope-correlation approaches and orthogonalization-based source-leakage corrections were described previously^[24,31]^. In this study, connectivity was calculated using AEC-c, and correlation coefficients were Fisher *z*-transformed before statistical testing.

Static connectivity was calculated over the 0–200-ms post-stimulus interval for each frequency band. Dynamic connectivity was estimated using 50-ms windows with 50% overlap (25-ms step) over 0–300 ms, yielding 11 windows centered at 25–275 ms. The six dynamic regions of interest were the left and right auditory cortices (A1L and A1R), frontal regions (FL and FR), and occipital regions (OL and OR).

Analyses were performed using MATLAB R2020b (The MathWorks, Inc., Natick, MA, USA) and Brainstorm (March 2025 version; Brainstorm development team)^[30]^. The supplementary network analysis used the NBS toolbox (version 1.2, 2012; Andrew Zalesky). The software environment also included BrainNet Viewer (version 1.7, release 20191031; Mingrui Xia, Beijing Normal University, Beijing, China). Early development used broadband (1–1000 Hz) signals for rapid identification of regions of interest to reduce computational load (***Supplementary Figs. 1*** and ***2*** and ***Supplementary Table 2***). The main text reports the complete band-specific analyses performed on the university's high-performance computing cluster; the supplementary broadband NBS component contained 24 unique cortical nodes, of which 22 were also involved in at least one significant frequency-specific static connection. The descriptive node-level recovery proportion was therefore 22/24 × 100% = 91.7%.

An exploratory, data-driven region of interest (ROI)-based dynamic functional connectivity analysis was subsequently performed using six regions informed by the static connectivity findings and exploratory broadband screening: the bilateral frontal lobes (FL and FR), bilateral primary auditory cortices (A1L and A1R), and bilateral occipital lobes (OL and OR). These regions were chosen because they are key nodes involved in auditory perception, top-down attentional control, and cross-modal integration networks—domains known to be disrupted in migraine. Focusing on these areas provided an overview of the temporal changes and hemispheric asymmetry of abnormal auditory-frontal-occipital interactions in migraine.

### Statistical analysis

Group differences in the primary static and dynamic connectivity analyses were assessed using two-sided independent-samples equal-variance *t*-tests, with 30 participants per group (*df* = 58). Static and dynamic analyses used separate multiple-comparison procedures.

For static connectivity, Bonferroni correction was applied separately within each stimulation condition across the valid off-diagonal elements of the 68 × 68 connectivity matrix and all seven frequency bands. Each family comprised 68 × 67 × 7 = 31892 tests, corresponding to an unadjusted *P* threshold of approximately 1.56779 × 10^−6^. Undirected connections are reported once per frequency band and stimulation condition.

For dynamic connectivity, false discovery rate correction was applied separately for each stimulation side across 6 × 5 × 11 × 7 = 2310 off-diagonal tests. Benjamini–Hochberg adjustment reconstructed from the saved *P* values at a level of 0.05 yielded rank-based unadjusted *P* boundaries of approximately 0.000995671 for right-ear stimulation and 0.000173160 for left-ear stimulation. Successive windows were treated as separate testing dimensions rather than grouped into temporal clusters. The figure-based annotation table identifies the values displayed in the retained graphics; it does not replace the independently audited statistical output.

Cohen's *d* was calculated from the *t* statistic and sample sizes. Where magnitudes are reported, |*d*| = |*t*| × √(1/*n*_1_ + 1/*n*_2_), with effect direction specified separately. The static table uses unrounded saved *t* values. Effect-size conversions made solely from displayed dynamic *t* values are explicitly identified in the annotation table.

A separate supplementary analysis used the NBS^[32]^. The analysis used a matched-pair general linear model with 30 pair-specific effects and corresponding exchangeability blocks. A primary threshold of *t* = 3.1 and component extent were used, with 5000 constrained permutations. One significant connected component comprising 24 nodes and 45 edges was identified (*P*_family-wise error rate_ = 0.0184). This result is reported as a supplementary matched-pair exploratory analysis. The component-level *P* value was not subjected to additional false discovery rate correction.

## Results

### Clinical characteristics

Of the 30 patients with migraine, 22 (73.33%) were female, and eight (26.67%) were male. Twenty-five patients (83.33%) experienced photophobia during an attack, and 26 (86.67%) reported phonophobia. Twenty-one patients (70.00%) had unilateral headaches. The average disease duration was 9.92 (± 5.72) years, and the average attack frequency was 2.91 (± 1.58) times per month. The clinical and demographic characteristics of patients with migraine, including sex, age, attack frequency, duration of illness, headache severity, and accompanying symptoms, are summarized in ***Table 1***.

**Table 1:** Summary of clinical and demographic characteristics of patients with migraine

Characteristics	Migraine	Control
Sex	Female/male: 22/8	Female/male: 22/8
Age (years)	31.07 ± 6.55	31.42 ± 6.72
Attack frequency (times/month)	2.91 ± 1.58	NA
Disease duration (years)	9.92 ± 5.72	NA
Headache severity (VAS 0–10)	5.47 ± 1.06	NA
Accompanying symptoms (cases)		
Photophobia	25	NA
Phonophobia	26	NA
Unilateral headache	21	NA
Bilateral headache	9	NA
Headache type (multiple selections, cases)		
Throbbing	18	NA
Dull pain	10	NA
Squeezing	5	NA
Stabbing	1	NA
Other	1	NA
Handedness	Right-handed: 30	Right-handed: 30
Continuous variables are expressed as mean ± standard deviation. Abbreviations: NA, not applicable (these variables were recorded in patients with migraine only); VAS, visual analog scale.		

### Static functional connectivity

Static functional connectivity differences surviving Bonferroni correction were confined to the low-gamma, high-gamma, and ripple bands. No connections survived correction in the delta, theta, alpha, or beta bands under either stimulation condition. ***Figs. 1*** and ***2*** illustrate a subset of these significant connections. ***Table 2*** reports the corresponding statistics for the connections illustrated in ***Figs. 1*** and ***2***, rather than an exhaustive set of all retained connections. All illustrated connections showed higher connectivity in patients with migraine than in healthy controls (Cohen's *d* = 1.40–1.48).

**Figure 1:**
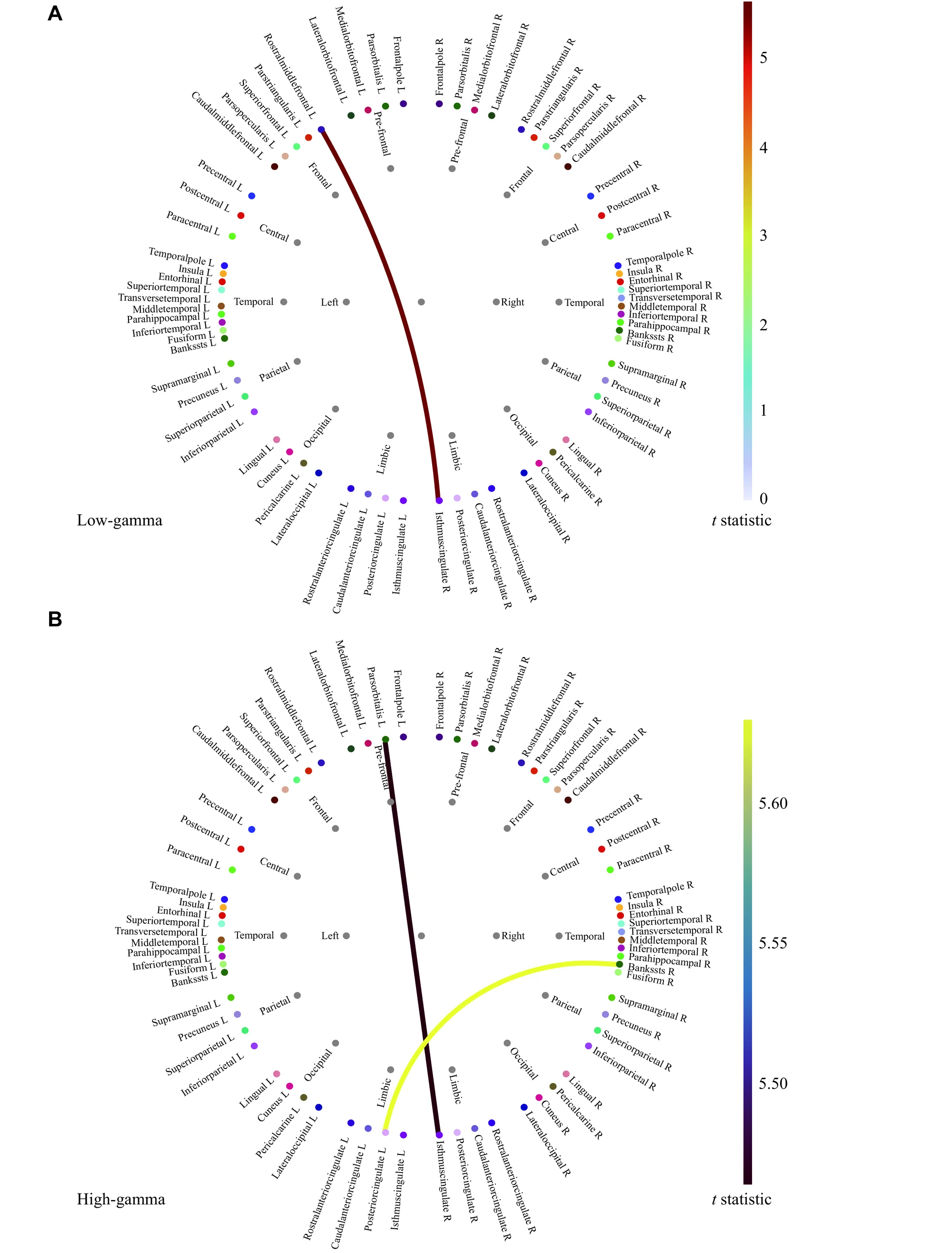
Static functional connectivity differences between patients with migraine and healthy controls following left-ear auditory stimulation (0–200 ms). A: A low-gamma connection (30–59 Hz) between the left rostral middle frontal gyrus (rostralmiddlefrontal L) and the right isthmus cingulate (isthmuscingulate R). B: Two high-gamma connections (60–89 Hz) between the left pars orbitalis (parsorbitalis L) and isthmuscingulate R, and between the right banks of the superior temporal sulcus (bankssts R) and the left posterior cingulate (posteriorcingulate L). All illustrated connections are interhemispheric and indicate higher connectivity in patients with migraine. The illustrated connections survived Bonferroni correction across 31892 tests within the stimulation condition; corresponding *t* statistics and effect sizes are reported in ***Table 2***. Abbreviations: L, left hemisphere; R, right hemisphere.

**Figure 2:**
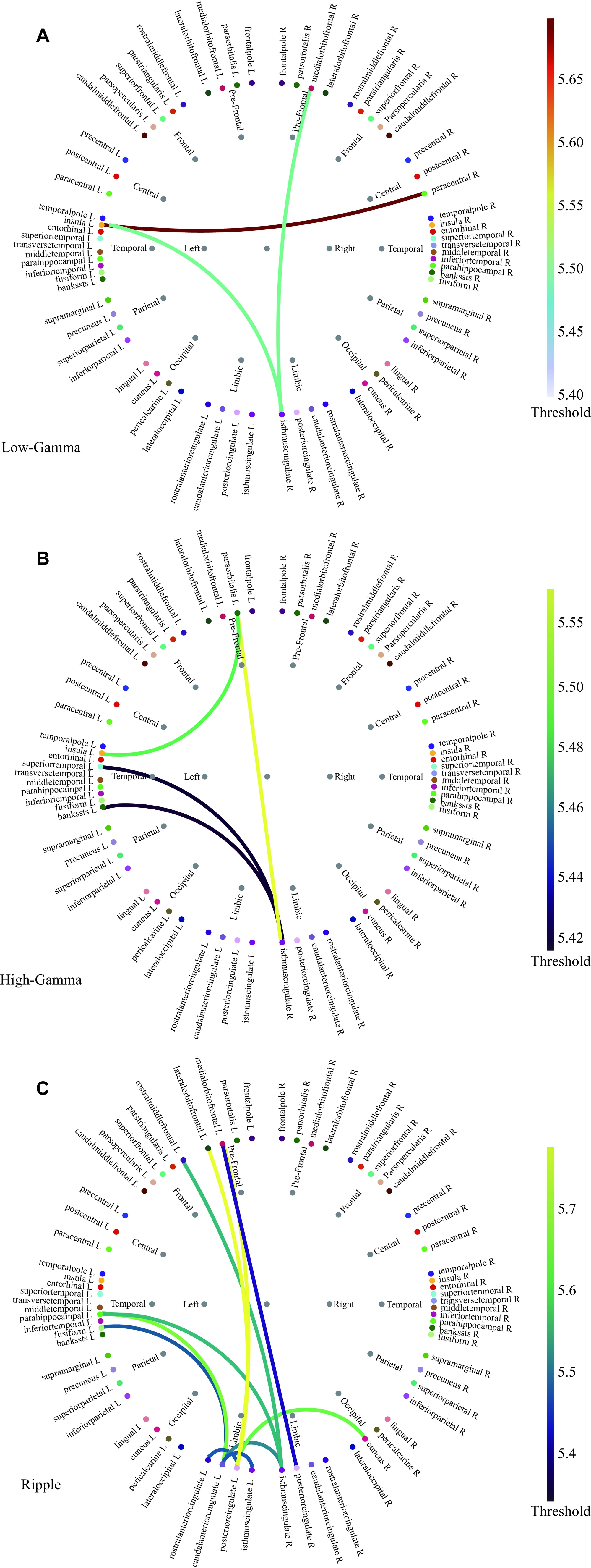
Static functional connectivity differences between patients with migraine and healthy controls following right-ear auditory stimulation (0–200 ms). A: Three low-gamma connections (30–59 Hz): insula L–paracentral R, insula L–isthmuscingulate R, and medialorbitofrontal R–isthmuscingulate R. B: Four high-gamma connections (60–89 Hz): bankssts L–isthmuscingulate R, superiortemporal L–isthmuscingulate R, parsorbitalis L–isthmuscingulate R, and insula L–parsorbitalis L. C: Eleven ripple-band connections (90–120 Hz) involving cingulate, orbitofrontal, rostral middle frontal, parahippocampal, fusiform, and cuneus regions; individual pairs are listed in ***Table 2***. All illustrated connections indicate higher connectivity in patients with migraine and survived Bonferroni correction across 31892 tests within the stimulation condition. This figure displays a subset of the significant connections. Abbreviations: L, left hemisphere; R, right hemisphere.

**Table 2:** Static functional connectivity differences following left- and right-ear auditory stimulation

Panels	Stimulation	Frequency band	Connection	Hemisphere	*t*	Cohen's *d*	Adjusted *P*
1A	Left ear	Low-gamma (30–59 Hz)	Isthmuscingulate R–rostralmiddlefrontal L	Interhemispheric	5.67	1.46	0.01490
1B	Left ear	High-gamma (60–89 Hz)	Bankssts R–posteriorcingulate L	Interhemispheric	5.63	1.45	0.01755
1B	Left ear	High-gamma (60–89 Hz)	Isthmuscingulate R–parsorbitalis L	Interhemispheric	5.46	1.41	0.03257
2A	Right ear	Low-gamma (30–59 Hz)	Insula L–isthmuscingulate R	Interhemispheric	5.53	1.43	0.02509
2A	Right ear	Low-gamma (30–59 Hz)	Insula L–paracentral R	Interhemispheric	5.70	1.47	0.01371
2A	Right ear	Low-gamma (30–59 Hz)	Isthmuscingulate R–medialorbitofrontal R	Right intrahemispheric	5.53	1.43	0.02567
2B	Right ear	High-gamma (60–89 Hz)	Bankssts L–isthmuscingulate R	Interhemispheric	5.41	1.40	0.04001
2B	Right ear	High-gamma (60–89 Hz)	Insula L–parsorbitalis L	Left intrahemispheric	5.50	1.42	0.02872
2B	Right ear	High-gamma (60–89 Hz)	Isthmuscingulate R–parsorbitalis L	Interhemispheric	5.53	1.43	0.02531
2B	Right ear	High-gamma (60–89 Hz)	Isthmuscingulate R–superiortemporal L	Interhemispheric	5.41	1.40	0.03984
2C	Right ear	Ripple (90–120 Hz)	Caudalanteriorcingulate L–fusiform L	Left intrahemispheric	5.50	1.42	0.02900
2C	Right ear	Ripple (90–120 Hz)	Caudalanteriorcingulate L–isthmuscingulate L	Left intrahemispheric	5.49	1.42	0.02928
2C	Right ear	Ripple (90–120 Hz)	Caudalanteriorcingulate L–isthmuscingulate R	Interhemispheric	5.54	1.43	0.02467
2C	Right ear	Ripple (90–120 Hz)	Caudalanteriorcingulate L–parahippocampal L	Left intrahemispheric	5.68	1.47	0.01476
2C	Right ear	Ripple (90–120 Hz)	Cuneus R–posteriorcingulate L	Interhemispheric	5.66	1.46	0.01568
2C	Right ear	Ripple (90–120 Hz)	Isthmuscingulate R–parahippocampal L	Interhemispheric	5.59	1.44	0.02072
2C	Right ear	Ripple (90–120 Hz)	Isthmuscingulate R–rostralmiddlefrontal L	Interhemispheric	5.58	1.44	0.02088
2C	Right ear	Ripple (90–120 Hz)	Lateralorbitofrontal L–posteriorcingulate L	Left intrahemispheric	5.72	1.48	0.01251
2C	Right ear	Ripple (90–120 Hz)	Medialorbitofrontal L–posteriorcingulate L	Left intrahemispheric	5.74	1.48	0.01146
2C	Right ear	Ripple (90–120 Hz)	Medialorbitofrontal L–posteriorcingulate R	Interhemispheric	5.44	1.40	0.03586
2C	Right ear	Ripple (90–120 Hz)	Posteriorcingulate L–rostralanteriorcingulate L	Left intrahemispheric	5.52	1.43	0.02650
The listed connections represent the subset illustrated in ***Figs. 1*** and ***2***, with higher connectivity in patients with migraine than in healthy controls. Statistics were obtained over the 0–200-ms post-stimulus interval using two-sided independent-samples equal-variance *t*-tests (30 participants per group; *df* = 58). *P* values are Bonferroni-adjusted across 31892 tests separately for each stimulation condition. Each undirected connection is listed once per frequency band and stimulation side. Cohen's *d* was calculated from unrounded *t* statistics as *d* = *t* × √(1/*n*_1_ + 1/*n*_2_), where *n*_1_ = *n*_2_ = 30. Interhemispheric connections link the left and right hemispheres; left and right intrahemispheric connections link regions within the corresponding hemisphere. Region labels follow the Desikan–Killiany atlas. L, left hemisphere; R, right hemisphere.							

Following left-ear stimulation, ***Fig. 1A *** shows increased low-gamma connectivity between the left rostral middle frontal gyrus and the right isthmus cingulate (*t* = 5.67, *d* = 1.46). ***Fig. 1B *** shows two high-gamma connections: the left pars orbitalis–right isthmus cingulate connection (*t* = 5.46, *d* = 1.41), and the right banks of the superior temporal sulcus–left posterior cingulate connection (*t* = 5.63, *d* = 1.45). All three connections are interhemispheric.

Following right-ear stimulation, ***Fig. 2A*** shows three low-gamma connections linking the left insula to the right paracentral lobule and the right isthmus cingulate, and the right medial orbitofrontal cortex to the right isthmus cingulate (*t* = 5.53–5.70, *d* = 1.43–1.47). ***Fig. 2B*** shows four high-gamma connections linking the right isthmus cingulate to the left banks of the superior temporal sulcus, left pars orbitalis, and left superior temporal gyrus, together with a connection between the left insula and left pars orbitalis (*t* = 5.41–5.53, *d* = 1.40–1.43). ***Fig. 2C*** shows 11 ripple-band connections involving cingulate, orbitofrontal, rostral middle frontal, parahippocampal, fusiform, and cuneus regions (*t* = 5.44–5.74, *d* = 1.40–1.48). Exact pairs and statistics for the illustrated connections are provided in ***Table 2***.

### Dynamic functional connectivity

***Figs. 3*** and ***4*** present the dynamic connectivity plots following left- and right-ear stimulation. In the left-ear plot, the delta-labeled panel depicts FL–OL at 25 ms (displayed *t* = −4.34) and FR–OL at 225 ms (displayed *t* = −4.21). The negative annotations indicate reductions rather than early delta enhancement. The beta-labeled panel depicts A1R–OL with an annotation of 225 ms (*t* = −4.28), whereas the ripple-labeled panel depicts A1L–FR at 50 ms (*t* = 4.35).

**Figure 3:**
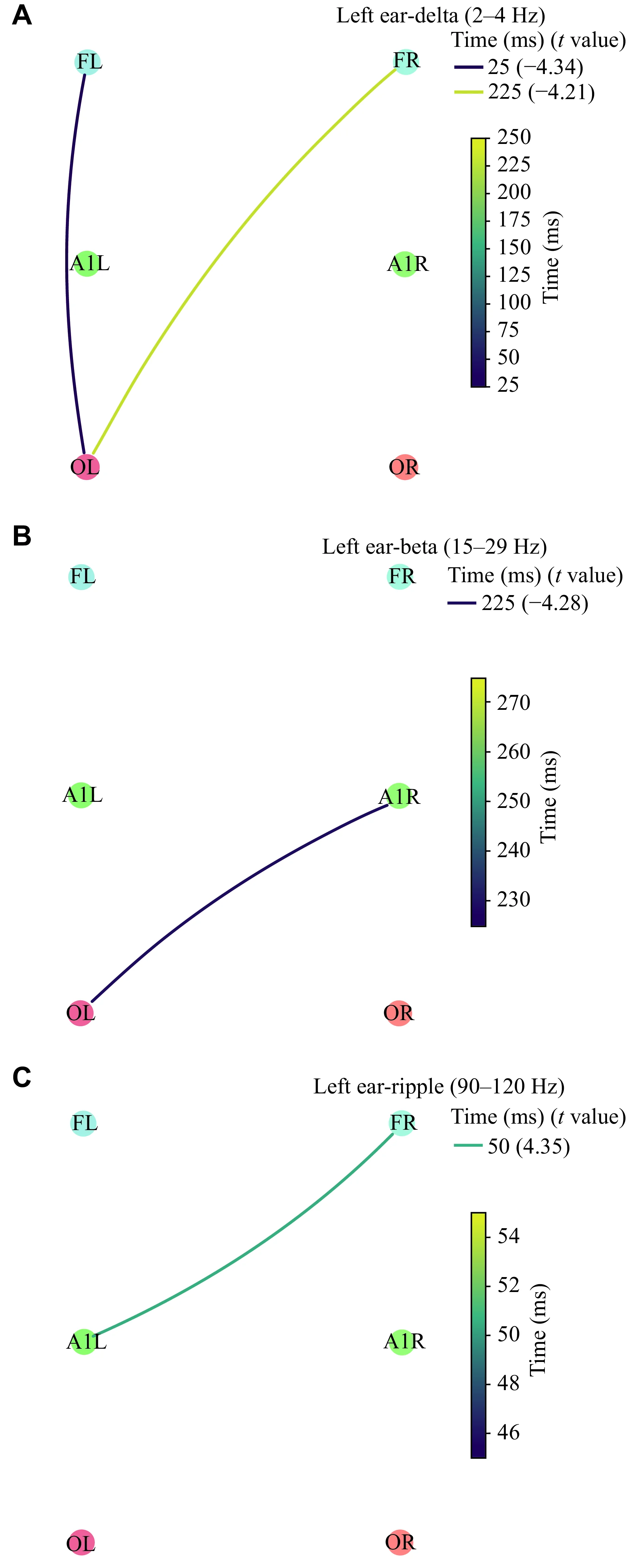
Dynamic functional connectivity plots following left-ear auditory stimulation. A: The panel labeled delta (2–4 Hz) depicts FL–OL and FR–OL connections, annotated as 25 ms (*t* = −4.34) and 225 ms (*t* = −4.21), respectively. B: The panel labeled beta (15–29 Hz) depicts an A1R–OL connection annotated as 225 ms (*t* = −4.28). C: The panel labeled ripple (90–120 Hz) depicts an A1L–FR connection annotated as 50 ms (*t* = 4.35). Negative and positive *t* annotations denote the displayed decreases and increases, respectively, for the patient-versus-control comparison. Abbreviations: A1L/A1R, left/right auditory cortex; FL/FR, left/right frontal lobe; OL/OR, left/right occipital lobe.

**Figure 4:**
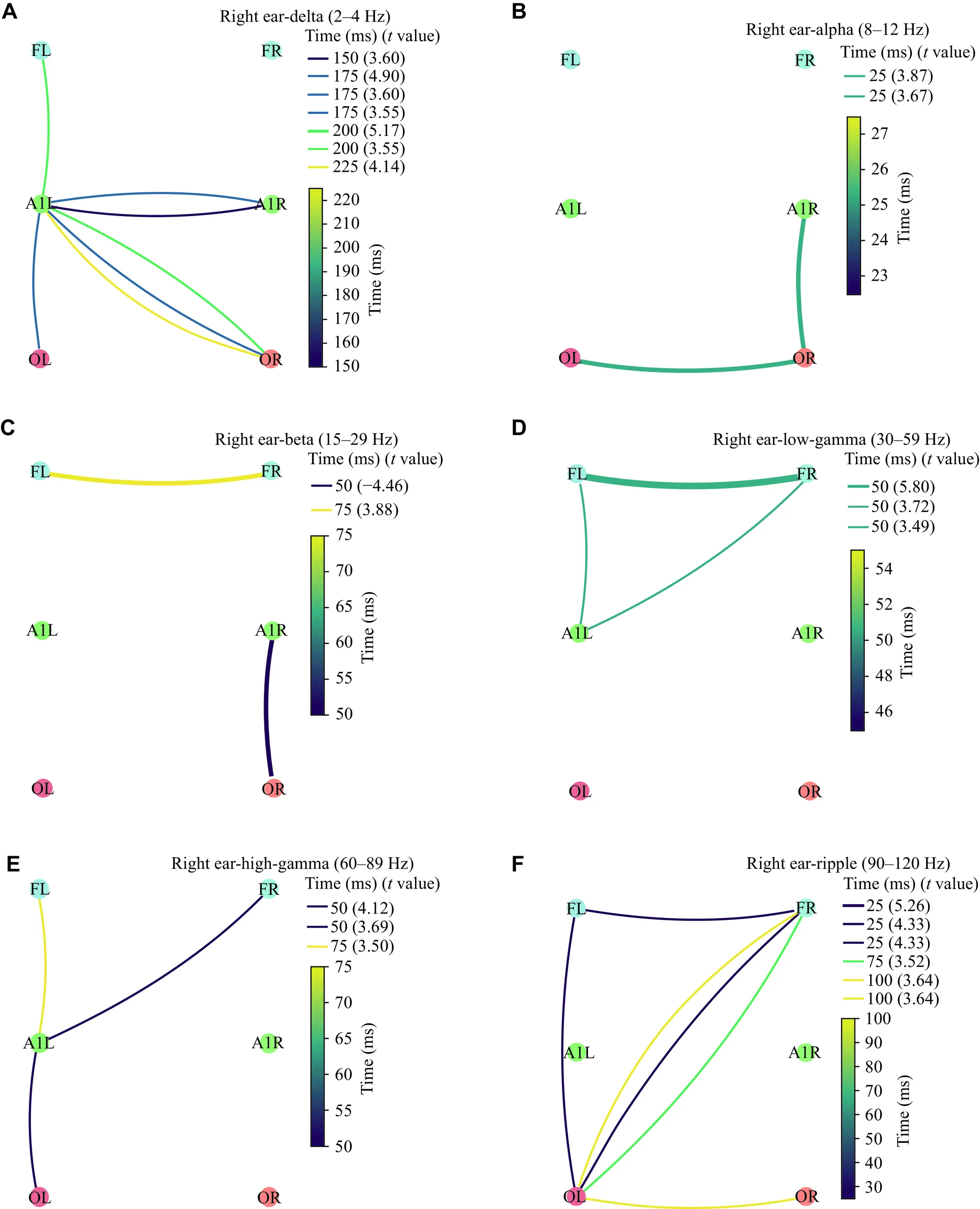
Dynamic functional connectivity patterns following right-ear auditory stimulation. A: The panel labeled delta (2–4 Hz) depicts connections linking A1L with A1R, FL, OL, and OR. Legend annotations span 150–225 ms, with displayed *t* values ranging from 3.55 to 5.17. B: The panel labeled alpha (8–12 Hz) depicts A1R–OR and OL–OR connections at 25 ms, with *t* = 3.67 and *t* = 3.87, respectively. C: The panel labeled beta (15–29 Hz) depicts an A1R–OR connection annotated as 50 ms (*t* = −4.46) and an FL–FR connection annotated as 75 ms (*t* = 3.88). D: The panel labeled low-gamma (30–59 Hz) depicts FL–FR, A1L–FR, and A1L–FL connections; all three legend entries are labeled 50 ms, with displayed t values of 5.80, 3.72, and 3.49. E: The panel labeled high-gamma (60–89 Hz) depicts A1L–FR, A1L–OL, and A1L–FL connections, with legend entries at 50 ms (*t* = 4.12 and 3.69) and 75 ms (*t* = 3.50). F: The panel labeled ripple (90–120 Hz) depicts FL–FR, FL–OL, FR–OL, and OL–OR connections, including multiple curves between FR and OL. Its legend lists 25 ms (*t* = 5.26, 4.33, and 4.33), 75 ms (*t* = 3.52), and two entries at 100 ms (*t* = 3.64 for FR–OL and OL–OR). Negative and positive *t* statistics denote the displayed decreases and increases, respectively. Abbreviations: A1L/A1R, left/right auditory cortex; FL/FR, left/right frontal lobe; OL/OR, left/right occipital lobe.

In the right-ear plot, the delta-labeled panel depicts A1L connections with A1R, FL, OL, and OR, with legend entries spanning 150–225 ms. The alpha-labeled panel shows A1R–OR and OL–OR at 25 ms, with displayed *t* values of 3.67 and 3.87, respectively. The beta-labeled panel shows A1R–OR at 50 ms (displayed *t* = −4.46) and FL–FR at 75 ms (displayed *t* = 3.88). The low-gamma-labeled panel depicts FL–FR, A1L–FR, and A1L–FL, all annotated at 50 ms. The high-gamma-labeled panel depicts A1L–FR and A1L–OL with 50-ms annotations and A1L–FL with a 75-ms annotation. The ripple-labeled panel shows FL–FR, FL–OL, FR–OL, and OL–OR, with multiple curves between FR and OL. Its legend includes three entries at 25 ms, one at 75 ms, and two at 100 ms. The two 100-ms entries correspond to FR–OL and OL–OR, both with displayed *t* = 3.64. ***Table 3*** lists the corresponding annotations.

**Table 3:** Dynamic functional connectivity patterns following left- and right-ear auditory stimulation

Panels	Stimulation	Frequency band	Connection	Time (ms)	*t*	Cohen's *d*	Direction
3A	Left ear	Delta (2–4 Hz)	FL–OL	25	−4.34	1.12	Decrease
3A	Left ear	Delta (2–4 Hz)	FR–OL	225	−4.21	1.09	Decrease
3B	Left ear	Beta (15–29 Hz)	A1R–OL	225	−4.28	1.11	Decrease
3C	Left ear	Ripple (90–120 Hz)	A1L–FR	50	4.35	1.12	Increase
4A	Right ear	Delta (2–4 Hz)	A1L–A1R	150	3.60	0.93	Increase
4A	Right ear	Delta (2–4 Hz)	A1L–OR	175	4.90	1.27	Increase
4A	Right ear	Delta (2–4 Hz)	A1L–OL	175	3.60	0.93	Increase
4A	Right ear	Delta (2–4 Hz)	A1L–A1R	175	3.55	0.92	Increase
4A	Right ear	Delta (2–4 Hz)	A1L–OR	200	5.17	1.33	Increase
4A	Right ear	Delta (2–4 Hz)	A1L–FL	200	3.55	0.92	Increase
4A	Right ear	Delta (2–4 Hz)	A1L–OR	225	4.14	1.07	Increase
4B	Right ear	Alpha (8–12 Hz)	A1R–OR	25	3.67	0.95	Increase
4B	Right ear	Alpha (8–12 Hz)	OL–OR	25	3.87	1.00	Increase
4C	Right ear	Beta (15–29 Hz)	A1R–OR	50	−4.46	1.15	Decrease
4C	Right ear	Beta (15–29 Hz)	FL–FR	75	3.88	1.00	Increase
4D	Right ear	Low-gamma (30–59 Hz)	FL–FR	50	5.80	1.50	Increase
4D	Right ear	Low-gamma (30–59 Hz)	A1L–FR	50	3.72	0.96	Increase
4D	Right ear	Low-gamma (30–59 Hz)	A1L–FL	50	3.49	0.90	Increase
4E	Right ear	High-gamma (60–89 Hz)	A1L–FR	50	4.12	1.06	Increase
4E	Right ear	High-gamma (60–89 Hz)	A1L–OL	50	3.69	0.95	Increase
4E	Right ear	High-gamma (60–89 Hz)	A1L–FL	75	3.50	0.90	Increase
4F	Right ear	Ripple (90–120 Hz)	FL–FR	25	5.26	1.36	Increase
4F	Right ear	Ripple (90–120 Hz)	FR–OL	25	4.33	1.12	Increase
4F	Right ear	Ripple (90–120 Hz)	FL–OL	25	4.33	1.12	Increase
4F	Right ear	Ripple (90–120 Hz)	FR–OL	75	3.52	0.91	Increase
4F	Right ear	Ripple (90–120 Hz)	FR–OL	100	3.64	0.94	Increase
4F	Right ear	Ripple (90–120 Hz)	OL–OR	100	3.64	0.94	Increase
Connection labels, times, and *t* statistics correspond to the annotations in ***Figs. 3*** and ***4***. Effect sizes are reported as magnitudes, |*d*| = |*t*| × √(1/*n*_1_ + 1/*n*_2_), where *n*_1_ = *n*_2_ = 30. Effect-size magnitudes were derived from the displayed t statistics, except for the OL–OR ripple-band connection in panel 4F at 100 ms, for which the unrounded *t* statistic was used. Increase and decrease indicate higher and lower connectivity, respectively, in patients with migraine relative to healthy controls. This table provides a descriptive summary of the figure annotations rather than an independent assessment of statistical significance. A1L, left auditory cortex; A1R, right auditory cortex; FL, left frontal lobe; FR, right frontal lobe; OL, left occipital lobe; OR, right occipital lobe.							

## Discussion

### Overview and principal findings

This study used millisecond-scale MEG during the lateralized auditory task and revealed that interictal migraine is characterized by frequency-specific alterations in both static and dynamic functional connectivity. In the static analysis, significant group differences were confined to the low-gamma (30–59 Hz), high-gamma (60–89 Hz), and ripple (90–120 Hz) bands. No static connections survived correction in the delta, theta, alpha, or beta bands. Dynamic analyses revealed frequency- and stimulation-side-dependent temporal patterns, such as early gamma/ripple hyperconnectivity (25–50 ms), right-ear beta alterations at 50 and 75 ms, and a left-ear beta alteration at 225 ms. These findings indicate frequency- and latency-dependent connectivity alterations that vary with stimulation side^[19]^.

### Pathophysiological implications

The illustrated static differences involved frontal, temporal, cingulate, and occipital regions across the gamma and ripple bands. These findings may reflect altered coordination across auditory and association networks in migraine. Previous MEG studies have reported altered resting-state microstate patterns^[33]^ and abnormal neuromagnetic activity during visual stimulation^[34]^, providing broader context for altered brain activity in migraine.

The dynamic plots additionally depict connectivity changes that vary with frequency, latency, and stimulation side. However, the present connectivity differences alone do not establish excitatory-inhibitory imbalance, predictive-coding dysfunction, or a causal relationship with sensory symptoms. Compensatory or rebound explanations for later changes also remain hypotheses. These interpretations require direct experimental testing.

### Dynamic network mechanisms in migraine

AEC-c quantifies undirected amplitude-envelope covariation and cannot establish directional or causal interactions. The observed frequency- and latency-dependent patterns may be compatible with altered predictive processing; however, prediction, expectancy, and prediction error were not experimentally manipulated. This interpretation therefore remains speculative^[25]^.

### Clinical significance and biomarker potential

The observed patterns may provide candidate electrophysiological signatures for future studies of auditory processing in migraine. Related research has examined migraine classification using static and dynamic resting-state fMRI connectivity^[35]^, visual cortical excitability in patients with persistent visual aura using MEG^[36]^, and associations between dynamic EEG effective connectivity and fluctuating pain intensity in chronic migraine^[37]^. Noninvasive neuromodulation has also been investigated for migraine treatment^[38]^.

These studies address different modalities, populations, and outcomes and do not establish the clinical utility of the auditory connectivity patterns reported here. Diagnostic discrimination, prospective prediction, and treatment-response prediction were not evaluated in the present study. Independent validation and direct associations with phonophobia, auditory thresholds, and clinical outcomes are needed before considering diagnostic or therapeutic applications.

### Limitations and future directions

Although MEG offers unparalleled temporal resolution, source localization remains limited by inverse modeling assumptions and interindividual anatomical variability; future studies should incorporate individualized head models and multimodal imaging (*e.g.*, MRI-fMRI fusion). The relatively small MwA subgroup (*n* = 6) precluded robust subtype comparisons, and all recordings were interictal; extending the paradigm to peri-ictal or ictal phases would clarify whether these high-frequency anomalies precede or result from attacks^[19]^. Larger cohorts, longitudinal designs, and multisensory paradigms are needed to track network evolution and test causal relationships. These findings provide exploratory evidence of altered auditory functional connectivity in migraine; their clinical relevance requires further investigation and validation in larger, independent cohorts. Because ROI selection and dynamic analysis were performed in the same dataset, the dynamic findings should be considered exploratory and require replication using prospectively defined ROIs in an independent cohort. The dynamic analysis covered 0–300 ms using 50-ms windows. Some later windows extended beyond the earliest recorded button press at 268 ms, and motor preparation could also precede overt movement. Motor-related contributions to the dynamic findings, therefore, cannot be excluded. The static 0–200-ms interval preceded overt button presses, but this timing alone does not exclude premotor activity. Because connectivity measures were not directly correlated with phonophobia severity or auditory thresholds, the proposed relationship with auditory hypersensitivity remains inferential. Effect sizes derived from statistically selected peak connections may be upwardly biased because of peak-selection or winner's-curse effects and should be interpreted cautiously.

### Conclusions

MEG revealed differences in auditory functional connectivity between interictal patients with migraine and healthy controls. Static differences surviving correction were confined to gamma and ripple bands, while the dynamic findings indicated temporally varying network alterations. These observations motivate further study of auditory network organization in migraine. Their mechanistic and clinical significance requires independent replication and direct evaluation against sensory and clinical measures.

## Additional information

The online version contains supplementary material available at http://www.jbr-pub.org.cn/article/doi/10.7555/JBR.39.20250487.
