# Supplementary material for: Aberrant static and dynamic functional connectivity of auditory processing in migraine: A millisecond-scale magnetoencephalography study
Source: J Biomed Res. 2026 Sep 28;40(5):521–35. doi: 10.7555/JBR.39.20250487 (PMC13624468; doi:10.7555/JBR.39.20250487)
Supplement: Supplementary file 1 [file jbr-40-5-521-S1.pdf]

Available online at www.jbr-pub.org.cn

Open Access at PubMed Central

Journal of Biomedical Research, 2026 40(5): 1–3

Supplementary Data

JBR

Aberrant static and dynamic functional connectivity of auditory processing in migraine: A millisecond-scale magnetoencephalography study

Hongxing Liu[1], Yuanwen Yu[1], Di Wu[1], Qiqi Chen[2], Yingfan Wang[1], Minghao Li[1], Xiaoshan Wang[1,✉]

[1]*Department of Neurology, the Affiliated Brain Hospital of Nanjing Medical University, Nanjing, Jiangsu 210029, China;*
[2]*MEG Center, the Affiliated Brain Hospital of Nanjing Medical University, Nanjing, Jiangsu 210029, China.*

Supplementary broadband network-based statistic (NBS) analysis

A separate exploratory broadband NBS analysis was performed using individual 1–1 000-Hz connectivity matrices following right-ear stimulation. The saved analysis used a matched-pair general linear model with 30 pair-specific effects and corresponding exchangeability blocks. A primary threshold of t = 3.1 and component extent were used, with 5 000 constrained permutations. One significant connected network component comprising 24 nodes and 45 edges was identified ($P_{\text{family-wise error rate}}$ = 0.018 4), showing greater broadband connectivity in patients with migraine. The component-level P value was not subjected to additional FDR correction. Because the saved model used pair-specific effects and exchangeability blocks, this result is reported as a supplementary matched-pair exploratory analysis.

Supplementary Table 1 **Reaction times in the lateralized auditory task.**

Groups	*n*	Mean ± SD (ms)	Range (ms)
Migraine patients	30	418 ± 91	282–689
Healthy controls	30	406 ± 82	268–612
All participants	60	412 ± 87	268–689

Data are expressed as mean ± standard deviation (SD). Independent-samples t-test between groups: t_{58} = 0.56, P = 0.58 (no significant difference). The minimum recorded reaction time was 268 ms. The 0–200-ms static analysis interval preceded overt button presses, but premotor activity cannot be excluded. The later portion of the 0–300-ms dynamic analysis interval may overlap with button presses. Accuracy was > 98% in both groups (no significant difference).

✉Corresponding author: Xiaoshan Wang, E-mail: lidou2005@126.com, ORCID: 0000-0003-3452-0402.

Received: 06 November 2025; Revised: 26 November 2025; Accepted: 26 November 2025; Available online: 29 November 2025; Published date: 28 September 2026

CLC number: R745, Document code: A

The authors reported no conflicts of interest.

This is an open access article under the Creative Commons Attribution (CC BY 4.0) license, which permits others to distribute, remix, adapt and build upon this work, for commercial use, provided the original work is properly cited.

© 2026 by Journal of Biomedical Research.

https://doi.org/10.7555/JBR.39.20250487

Supplementary Table 2 **Comparison of significant static functional connectivity findings between broadband and band-specific analyses (right-ear stimulation, 0–200 ms)**

Measures	Result
Significant broadband NBS component	24 nodes and 45 edges
NBS component-level significance	P_{FWER} = 0.018 4
NBS nodes also involved in frequency-specific static networks	22
Descriptive node-level overlap	22/24 × 100% = 91.7%

Let R_NBS denote the set of unique cortical nodes in the significant broadband NBS component and R_band denote the set of cortical nodes involved in at least one significant frequency-specific static connection. Node-level overlap was calculated as |R_NBS intersection R_band| / |R_NBS| × 100% = 22/24 × 100% = 91.7%. This value represents a descriptive recovery proportion rather than a Jaccard index, an independent validation, or an additional inferential test. The broadband NBS analysis and the primary frequency-specific static analysis were performed using data from the same participants. Abbreviations: FWER, family-wise error rate; NBS, network-based statistic.

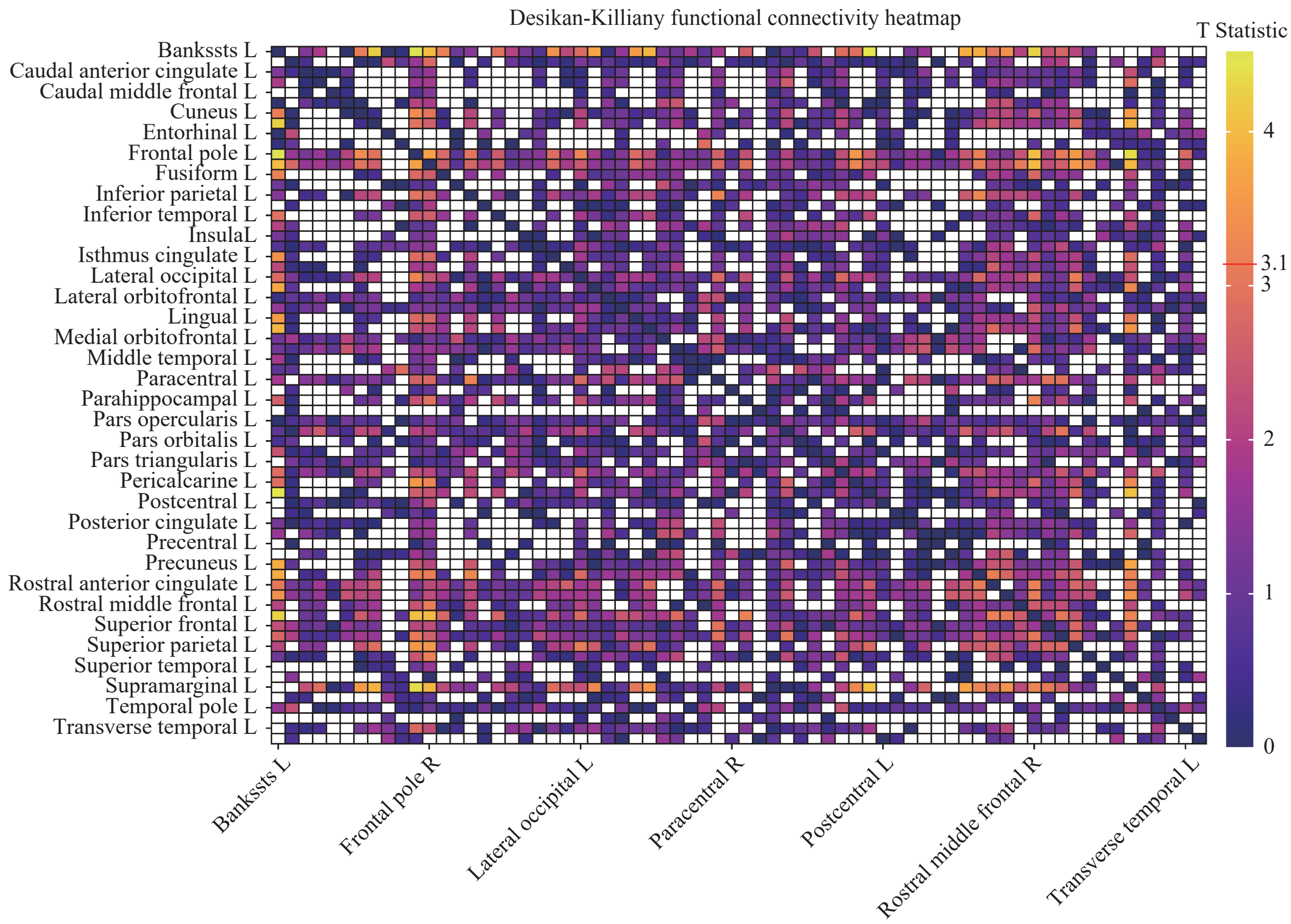

Supplementary Fig. 1 **Broadband functional-connectivity matrix across cortical regions following right-ear auditory stimulation.** This heatmap displays connection-wise t statistics using the Desikan–Killiany parcellation. The color bar represents the *t* statistic, and the red horizontal line marks the primary NBS threshold of t = 3.1.

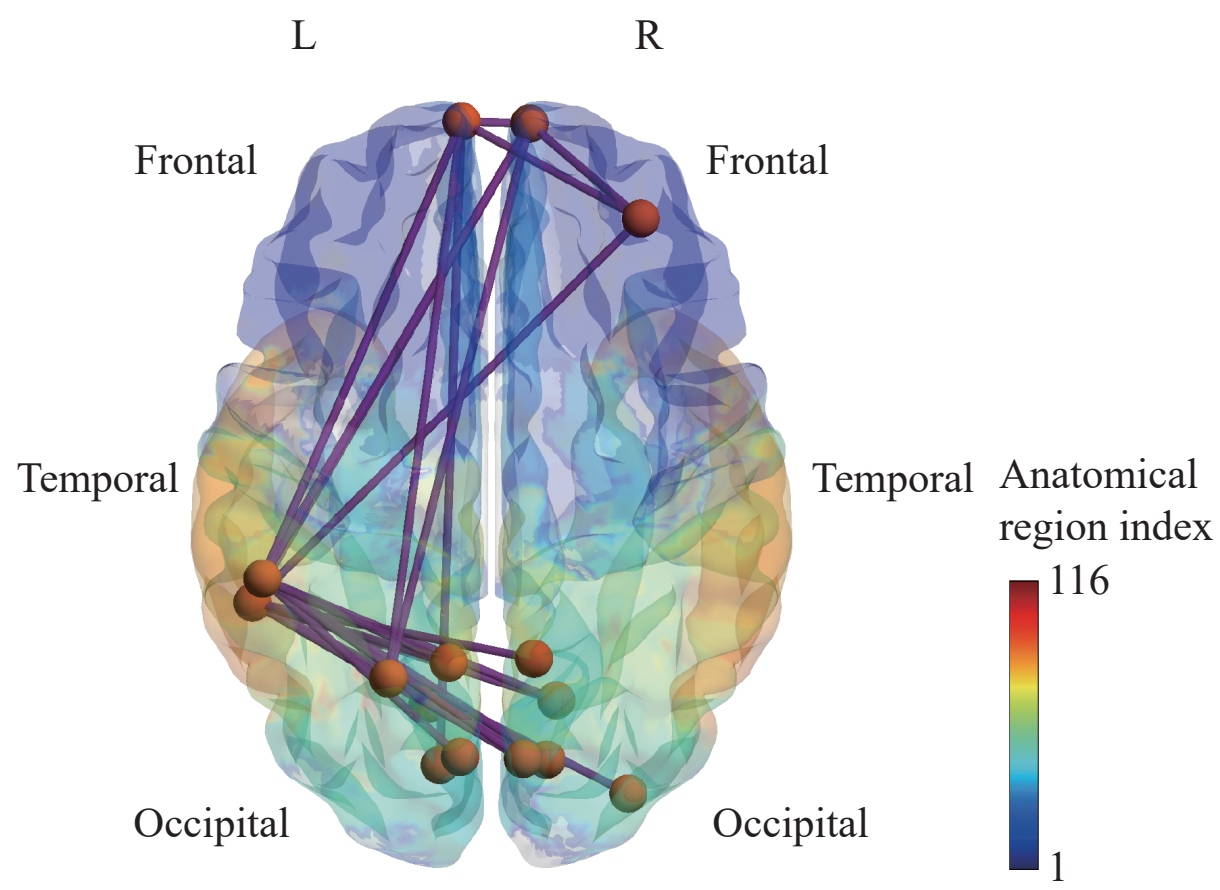

Supplementary Fig. 2 **Three-dimensional visualization of the supplementary broadband connectivity network following right-ear auditory stimulation.** The color bar represents the anatomical region index used for visualization and does not represent statistical significance. Node size reflects degree centrality, and line thickness reflects connection strength.
